# Supplementary material for: Deconvolving mutational patterns of poliovirus outbreaks reveals its intrinsic fitness landscape
Source: Nat Commun. 2020 Jan 17;11:377. doi: 10.1038/s41467-019-14174-2 (PMC6969152; doi:10.1038/s41467-019-14174-2)
Supplement: Supplementary file 1 — Supplementary Information [file 41467_2019_14174_MOESM1_ESM.pdf]

Supplementary Information

Quadeer et al. “Deconvolving mutational patterns of poliovirus outbreaks reveals its intrinsic fitness landscape”

Table of contents

Supplementary Notes 3

Supplementary Note 1. One-point correlations based maximum entropy model 3

Supplementary Note 2. Observed local peaks are not the result of finite sampling 3

Supplementary Note 3. Robustness to the regularization used in the landscape inference 3

Supplementary Note 4. Robustness to sequence reweighting used in the landscape inference 3

Supplementary Note 5. Details of peaks associated with polio outbreaks due to large gaps in immunization or incomplete immunization dosage 4

Supplementary Note 6. Analysis of peak 7 of the fitness landscape 5

Supplementary Note 7. Details of the experimental fitness measurements selected from literature for model validation 6

Supplementary Note 8. Comparative analysis based on the dN/dS metric 7

Supplementary Note 9. Interpretation and significance of model couplings 8

Supplementary Figures 10

Supplementary Tables 25

Supplementary References 28

Supplementary Notes

Supplementary Note 1. One-point correlations based maximum entropy model

For a maximum entropy model based on only one-point correlations, the model parameters $h_i(x)$ obey the explicit equation

$$h_i(x) = \log \frac{p_i(x)}{1 - p_i(x)}$$

where $p_i(x)$ is the frequency of observing a mutant x at residue i (see Methods, equation 3).

Supplementary Note 2. Observed local peaks are not the result of finite sampling

Finite sampling (due to the limited number of available sequences) can cause the model to infer non-zero couplings for mutants at independent pairs of residues, which can subsequently result in observing multiple peaks in the landscape. To test the contribution of sampling in the inferred vp1 landscape, we constructed a null case by shuffling the amino acids in each column of the MSA such that the number of amino acids observed at each residue remains the same, but the amino acids observed at different residues become uncorrelated[1]. We fitted the Potts model to infer the landscape for this shuffled case using the same procedure as discussed in Methods. In this case, only *one* local peak was observed in the inferred landscape, indicating that the multiple local peaks that we observe in the vp1 landscape are not an artifact of finite sampling.

Supplementary Note 3. Robustness to the regularization used in the landscape inference

Inferring a landscape from finite sample data involves the use of regularization, which can affect the local peaks observed in the landscape. In our landscape inference method, we used a L_2-norm regularization parameter which was set to the recommended value of $\frac{1}{M}$, where M is the number of available PV sequences[2]. To test the robustness of the observed peaks to the value used for regularization parameter, we re-inferred landscapes using a $\pm$ 50% change in the recommended value of the regularization parameter and found that the peaks remained almost the same (Supplementary Figure 12).

Supplementary Note 4. Robustness to sequence reweighting used in the landscape inference

In contrast to HIV sequence data, there is an additional sampling bias in the PV sequence data due to localized outbreaks during which a large number of similar sequences are sampled (e.g. the Congo 2010 and the Israel 2013 outbreaks). To compensate for this sampling bias, a reweighting scheme (similar to that used for reducing the bias due to multiple HIV sequences

per patient) can be employed that decreases the statistical weight of PV sequences that are genetically similar and correspond to the same country and the same year. (A similar procedure, albeit based purely on genetic distance, has been applied for maximum-entropy-based models to reduce the effect of sampling bias for predicting protein contacts[3,4].) With this sequence reweighting, the observed one- and two-point correlations of the MSA are given as

$$p_i(x) = \frac{1}{W_{\text{eff}}} \sum_{m=1}^{M} w_m \delta(s_i^m, x) \text{ and } p_{ij}(x,y) = \frac{1}{W_{\text{eff}}} \sum_{m=1}^{M} w_m \delta(s_i^m, x)\, \delta(s_j^m, y),$$

where $p_i(x)$ is the frequency of observing a mutant x at residue i, $p_{ij}(x,\ y)$ is the frequency of simultaneously observing a mutant x at residue i and a mutant y at residue j. The weight $w_m = 1/z_m$ compensates for the sampling bias, where z_m is the total number of genetically similar sequences (i.e., the sequences separated by a small Hamming distance D) in the MSA to the sequence m that are also obtained from the same country in the same year. The normalization factor $W_{\text{eff}} = \sum_{m=1}^{M} w_m$ is the effective number of sequences obtained after applying this weighting.

In order to assess the effect of such sequence reweighting, we applied this reweighting procedure for $D \leq d$ with $d \in \{0, 1, 2, 3\}$ and inferred new landscapes in each case. For each D, only minimal changes to our original inferred landscape were observed. Notably, the peak structure was very similar to our original landscape (Supplementary Figure 13a), with merging of a few specific peaks associated with largely immunized populations for larger d values, while peak 1, which represents natural PV evolution in largely unimmunized populations, remained virtually unaffected. Moreover, the fitness predictions based on the newly-inferred landscapes showed similar correlation with experimental fitness measurements as for the original landscape (Supplementary Figure 13b). This correlation is notably much lower than that obtained for the landscape based on only the peak 1 sequences, which we propose to be a meaningful representation of the PV fitness landscape. These results, in general, show that attempting to account for sampling bias by simply reweighting sequences does not sufficiently compensate for the vaccine-associated bias present in the PV sequence data.

Supplementary Note 5. Details of peaks associated with polio outbreaks due to large gaps in immunization or incomplete immunization dosage

Peaks 2 and 4 were found to primarily represent the outbreak of wild-type PV in an Egyptian population during 2000-2005, which occurred due to lapses in immunization[5]. Similarly, peak 3 represented the 2010 outbreak in the Republic of Congo, which mainly affected the immunized population[6]. This was confirmed by the presence of neutralizing antibody titers against all Sabin strains in the reported fatal cases. This outbreak had a rare (very high) mortality rate of 47% due to a mutation in the antigenic sites, which was able to evolve due to the compromised immunity of the population—a consequence of incomplete OPV dosage. While peak 6 appeared geographically well-mixed (Fig. 3a), it largely corresponded to (i) the 1988 outbreak in an immunized Israeli population with low antibody titers due to large lapses in immunization[7], and (ii) the 1980 outbreak in northern Andean region’s (Venezuela to Peru) immunized population due to importation of a variant wild-type from Middle East[8]. Similarly, peaks 8 and 9 consisted of a majority of sequences from the outbreaks in Ghana and other

neighboring North-western African during the early 2000s[9]. While these countries had been declared polio free in the late 1990s, subsequent unstable political conditions and shortage of vaccines hindered immunization activities in the region. The importation of wild-type PV from the neighboring endemic country, Nigeria, enabled the virus to cause outbreaks by infecting the partially immunized population of this region.

Supplementary Note 6. Analysis of peak 7 of the fitness landscape

The sequences in peak 7 of the inferred fitness landscape are associated with the circulation of PV in Pakistan and Afghanistan during 2010-2011. In contrast to other reported outbreak-associated peaks, there is no antigenic mutation in peak 7 (Fig. 2b). However, it is geographically and temporally localized (Fig. 3). Thus, it is not clear if this peak is associated to a specific outbreak, particularly because both Pakistan and Afghanistan are endemic countries. The report associated with the majority of sequences in this peak[10] appeared to be a random surveillance study of wild-type PV circulation near the Pakistan-Afghanistan border; suggesting that similar to peak 1, peak 7 may also represent the circulation of PV in unimmunized population. We tried to investigate this in detail using the available data as discussed below.

The phylogenetic tree (Supplementary Figure 5a) shows that peak 7 sequences spurred out of peak 1 sequences also belonging to South Asia (Pakistan and Afghanistan) (Supplementary Figure 5b, left panel) and thus, peak 7 may be an extension of peak 1. If this is true, we hypothesized that peak 7 should be closer to peak 1 as compared to other outbreak peaks. However, investigating the Hamming distances between the sequences in peak 7 and those in peak 1 did not show peak 7 to be the closest to peak 1 (Supplementary Figure 14a). It appeared to be the second-closest to peak 1, with peak 3 being the closest. We also tried to quantify the possible close proximity of peak 7 to peak 1 by running a zero-temperature MCMC simulation, which involved starting multiple trajectories from each sequence in peak 1 and determining the number of mutation steps required to reach other peaks. The smaller the number, the closer a peak would be to peak 1. The number of steps required to reach peak 7 was again found to be the second-smallest after peak 3 (Supplementary Figure 14b). Interestingly, peak 7 and peak 5 (representing Israel 2013 outbreak) get merged into a single peak if landscape is inferred by re-weighting of sequences for suppressing sampling bias in the data (Supplementary Figure 14a). This suggests that the sequences in peak 5 are closely related to those in peak 7, which is consistent with the fact that the wild-type PV that circulated in a well-immunized population of Israel in 2013 was imported from South Asia[11]. Thus, similar to peak 1, peak 7 may also be considered a reservoir of PV sequences in largely unimmunized populations that can be transmitted to cause outbreaks in regions with a sub-optimally immunized population (see Results for details).

Nevertheless, we also computed the correlations with experimental fitness values by considering both peaks 1 and 7 to be representative of the unimmunized population (Supplementary Figure 14c) and the remaining peaks to be representative of outbreaks (Supplementary Figure 14d). The correlation values for both cases (-0.82 and -0.50, respectively) remains almost the same as compared to Fig. 5a (-0.83) and Supplementary Figure 4b (-0.51), respectively.

Supplementary Note 7. Details of the experimental fitness measurements selected from literature for model validation

The experimental fitness values for PV were obtained after an extensive survey of studies[12–15] in the literature. As there are multiple experimental measurements reported in each study, we detail below the criteria we used to select the specific fitness measurements (listed in Supplementary Table 2) from each report[11–15].

In ref[15], the authors compared in Table I the growth (in PFU/ml) of Mahoney and soluble receptor-resistant (srr) mutants in the presence and absence of soluble poliovirus receptor (S100Pvr). As our model is trained based on *vp1 sequences* evolving in the population with the *wild-type Pvr*, we only selected the virus titers reported in the absence of S100Pvr. The srr mutants in the vp1 protein are listed in Table II. Note that as all mutants were found to have similar titers, only the average titer value for srr mutants was reported in Table I. Thus, for fair comparison, we also averaged the energies (predicted from our model) corresponding to each mutant strain. In Fig. 5a, only two data points are associated with this report: one for the Mahoney strain and the other for the averaged srr mutants' strains. The binding affinities and alteration ratios of srr mutants, reported in Tables III and IV respectively, were excluded from our analysis as we considered viral titers (Table I) to be a better representative of the viral replicative fitness.

In ref[12], the authors compared in Table 1 the growth (in PFU/ml) of wild-type and mutant PV (serotypes 1, 2, and 3) on cells expressing wild-type and mutant (d, g, and i) Pvr. As our model is trained based on *PV serotype 1 vp1 sequences* evolving in the population with the *wild-type Pvr*, we selected only the virus titers reported for Mahoney (serotype 1 wild-type PV) and the associated vp1 mutant strains on cells expressing the wild-type Pvr. Moreover, note that the measurement for the strain with P95S mutant was selected while that with P95T mutant was not because we only observed one mutation (P to S) at position 95 in the available vp1 sequence data. Also, the binding affinities reported in Table 3 were not used for the same reason as mentioned above for ref[15].

In ref[14], the virus titers of wild-type and mutant poliovirus determined by plaque assays on HeLa cell monolayers at 37 degrees Celsius as well as a slightly higher temperature of 40 degrees Celsius were reported in Table 2. These titers were reported for the Mahoney strain, the Sabin strain, multiple recombinant strains of Mahoney and Sabin strains, and multiple mutant Mahoney strains with mutations in different viral proteins. We only considered the eight virus titers related to our work, i.e., those corresponding to the Mahoney strain and its mutants in the vp1 protein (listed in Supplementary Table 2). We excluded the Sabin strain as the vaccine-related strains were not included in the data used for inferring our model (see Supplementary Figure 8). As virus titers at high temperature correlate better with data from human isolates, we included in our analysis the virus titers determined at higher temperature (40 degrees Celsius) in Table 2. Moreover, we did not include the 50% paralytic/lethal dose (PLD50) values reported in Table 1 of ref[14] as these did not seem to correlate well (Supplementary Figure 16a) with the reported titers for strains with the same mutations in Table 2 of ref[14].

In ref[13], the authors reported in Table 2 the growth (in PFU/ml) of (i) Δ9 strain: a B-C loop (residues 98–102) truncated Mahoney strain, (ii) 414 strain: Mahoney strain with B-C loop replaced with the corresponding sequence in the serotype 2 Lansing strain, and (iii) multiple variants of Δ9 and 414 strains that are adapted to three Pvr mutant cell lines (d, g, and i). All the virus titers reported for the latter multiple variants (Tables 4, 6, 7, and 8), except the variant Δ9-1/i, were not considered as these adapted viruses included mutations in proteins other than vp1 (shown in Table 3 of ref[13]). The variant Δ9-1/i included three mutants in vp1 and the growth of strains having all possible (seven) combinations of these mutants were studied by site-directed mutagenesis in Δ9 strain on d, g, i, and 20B cells in Table 5 of ref[13]. As in the case of ref[12] (mentioned above), we only considered the virus titers reported in Table 5 for 20B cells as these express wild-type Pvr. The titer for Δ9-1/i reported in Table 5 of ref[13] was not included as it is essentially similar to the titer reported for the Δ9 strain with all the three mutants. Moreover, we had previously excluded the 414 strain as it involved a part of the serotype 2 Lansing strain in vp1. However, we investigated further and found that our model predictions are robust to the inclusion of the virus titer of the 414 strain and the associated strain with mutation (E168G) in vp1 (reported in Table 2 and 8, respectively) in the analysis (Supplementary Figure 16b). Note that other virus titers reported in Table 8 could not be included as these involved mutations in proteins other than vp1.

In ref[11], the neurovirulence, in terms of 50% paralytic dose (PD50), of multiple strains in transgenic mice with wild-type Pvr was reported in Table 2. Of these strains, we did not include the measurements reported for the Sabin strain due to the same reason as mentioned above for ref[14]. The measurements reported for the strains 02v9529 and PAK-5388 were also excluded as the authors did not provide the accession numbers associated with them. (02v9529 appeared to be a vaccine-derived strain[16] and thus not related to our analysis). The remaining PD50 measurements reported in Table 2 of ref[11] for the Mahoney strain and the three Israel 2013 outbreak-associated strains were included in our analysis. Data reported in Table 3 of ref[11] was not included as it was related to neutralization potency of antisera from rats immunized with IPV.

Supplementary Note 8. Comparative analysis based on the dN/dS metric

For comparison, we conducted the standard dN/dS analysis for the considered PV and HIV proteins using the commonly employed MEGA7 software and selecting the well-known Li-Wu-Luo method [17]. Interestingly, this analysis would suggest that each protein is under similar level of purifying selection (average(dN-dS) < 0), and that that there is not much distinction between the evolutionary constraints faced by these proteins (Supplementary Figure 15). Similar qualitative results were obtained using another dN/dS analysis method—the modified Nei-Gojobori method.

We note that these dN/dS results may not be meaningful for comparing the studied proteins as the sequence data of each protein is obtained from a single viral population clade/serotype, and for such cases, as opposed to distantly diverged sequences, dN/dS analysis has been convincingly argued to be unsuitable for inferring positive or purifying selection[18]. In fact, for analysis within a population, it was shown that dN/dS is insensitive to the selection coefficient

and dN/dS>1 (or equivalently dN-dS>0), the widely-accepted signature of positive selection over divergent lineages, does not hold.

In contrast to dN/dS analysis, our work, which proposes to employ the fitness landscapes of PV and HIV for comparing the evolutionary constraints faced by these proteins appears to be more meaningful. A major reason is that the fitness landscapes have been demonstrated to be representative of the underlying evolutionary constraints by contrasting against experimental fitness measurements (HIV proteins in previous reports[19–24] and PV vp1 in this work). They also account for both site-wise variation and mutational interactions among residues.

Supplementary Note 9. Interpretation and significance of model couplings

We provide here the interpretation of the model couplings and potential biological meaning. There are two sets of couplings that should be considered: those in the original model based on all sequences, and those in the model based on peak-1 sequences only, which we propose as the PV fitness landscape model.

For the first (all-sequence) model, we recall that incorporating couplings using pairwise residue variation is *necessary* to observe the peak structure, since only a single peak is observed if a model is inferred using single-residue variation only (details in Supplementary Notes 1 and 2). Moreover, the analysis of the peak structure was crucial to disentangle the natural PV evolution from the vaccine-driven (outbreaks-associated) evolution, which then allowed us to suppress the strong sampling bias in the available PV sequence data caused by outbreaks and obtain a meaningful PV fitness landscape.

Examining the strongest couplings in the model, corresponding to the 1% of those with the highest magnitudes, reveals that each observed peak is enriched with such couplings. Many of these couplings also involve the known biologically-important vp1 *antigenic sites* (Supplementary Figure 17a). This is consistent with the suggested role of antigenic mutations in escaping the immune pressure and triggering outbreaks (Figs. 2b, 2c). This has been discussed in ref[6] in the context of the 2011 Congo outbreak (represented by peak 3 in our model), when antigenic mutations in the circulating strain resulted in a large number of fatalities.

More generally, the peak structure induced by the couplings appears to be a manifestation of biased correlations resulting from localized variation observed in specific outbreaks that is tied to antigenic variation. That is, sequences in outbreaks tend to be closely related, sharing highly specific sets of mutations. We conducted additional tests to demonstrate this, by showing that mutations in each “peak sequence” in the model (i.e., the most fit, or lowest energy, sequence in a peak), referred to as “peak mutations” henceforth, were shared by a large majority of sequences falling in the same peak, inducing strong localized mutational correlations (Supplementary Figure 17b). In contrast, other mutations in the sequences falling in the same peak, referred to as “non-peak mutations” here onwards, occurred at a much lower frequency and consequently induced much weaker correlations (Supplementary Figure 17b).

From an evolutionary perspective, the strong couplings observed among peak mutations may be seen to arise due to "genetic linkage". (That is, due to the over-representation of background variation coupled to antigenic variation offering a selective advantage.) Conceptually, this phenomenon is similar to that observed in the evolution of influenza virus[25,26], in which multiple neutral or deleterious mutations linked to antigenic mutations often collectively rise to fixation. To further quantify the effect of genetic linkage in shaping the outbreak-associated peaks, we computed the linkage disequilibrium[27] in the sequences associated with each peak using the standard Lewontin D' metric[28] (Supplementary Figure 17c). This analysis suggested that all of the top 10 peaks, except peak 1, were seemingly strongly affected by genetic linkage (mean D' ~ 1).

The coupling structure for the model built from sequences associated with largely unimmunized population (represented by peak 1) is different, and the landscape has only a single peak, as we have described in the main text. The majority of the residue pairs linked by strong couplings in this model were distinct from those in the all-sequence model (Supplementary Figure 18a), and there is no longer a strong enrichment of antigenic mutations in this set. The linkage disequilibrium in the sequences associated with peak 1 is also substantially less (Supplementary Figure 17c). This supports the notion that the second model is subject to significantly lower sequence bias than the all-sequence model, and this leads to a model which is a meaningful proxy for the vp1 fitness landscape, as we demonstrate through comparison with independent experimental fitness data (Fig. 5a).

The couplings in the model inferred from sequences in peak 1 only are statistically smaller in magnitude ($P = 10^{-32}$; two-sided Mann-Whitney test) as compared to those inferred in the all-sequence model (Supplementary Figure 18b). Note that this result is consistent with the known reduced role of epistasis in PV evolution[29,30]. However, investigating the top 1 percentile model couplings shows that these are enriched in N terminus vp1 residues which form an interface with the capsid vp4 protein (Fig. 5b) known to be critical for viral stability[31]. This suggests that the strong couplings in this model are reflective of inherent structural constraints. This result sheds direct light on the biological meaning of the intrinsic fitness landscape that we have inferred after carefully removing sampling biases due to localized outbreaks, vaccination, etc.

To summarize, the inferred couplings in the original model are vital for suppressing the strong sampling bias in the available PV sequence data caused by outbreaks, which then enabled us to obtain a meaningful PV fitness landscape. The strong couplings in this model are enriched in antigenic mutations which seemingly facilitate outbreaks. In contrast, the couplings in the model built from only peak 1 sequences (representing the PV fitness landscape) seem to be biologically informative from a structural/virus fitness perspective, and is consistent with the reported limited role of interactions between residues in PV evolution[29,30].

Supplementary Figures

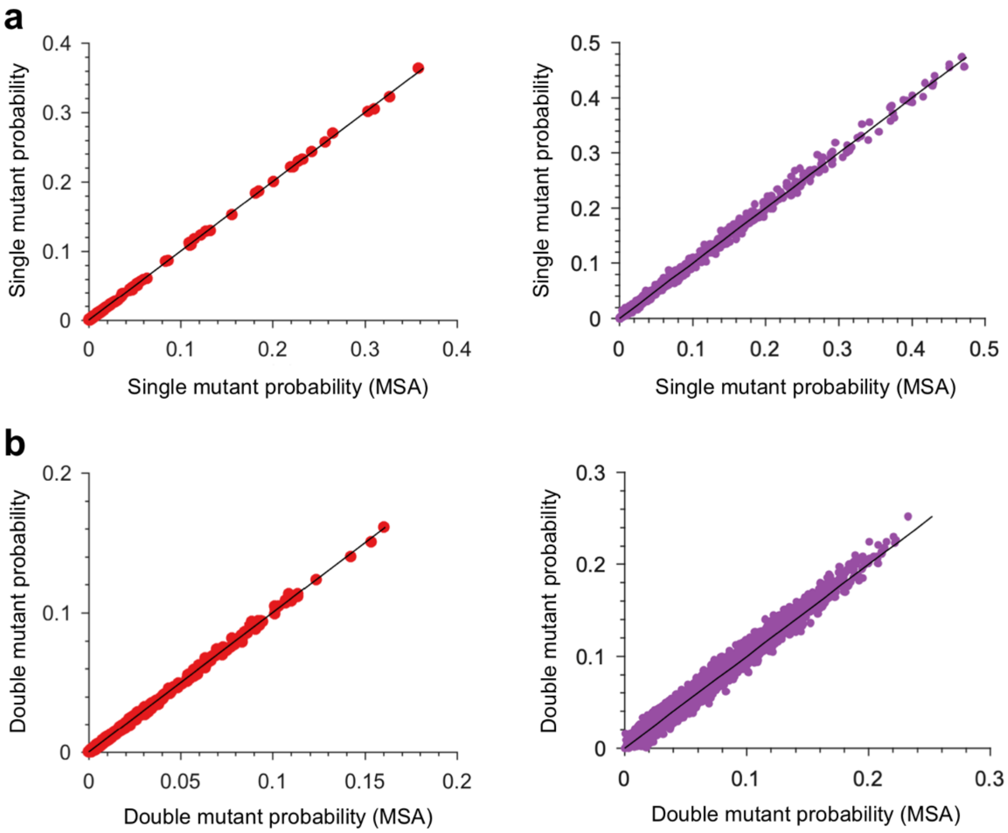

Supplementary Figure 1. The inferred Potts model of the p24 (red) and gp160 (purple) proteins is fairly accurate. Comparison of (**a**) the single mutant and (**b**) the double mutant probabilities of the MSA (used to train the model) and the sequences generated from the inferred model.

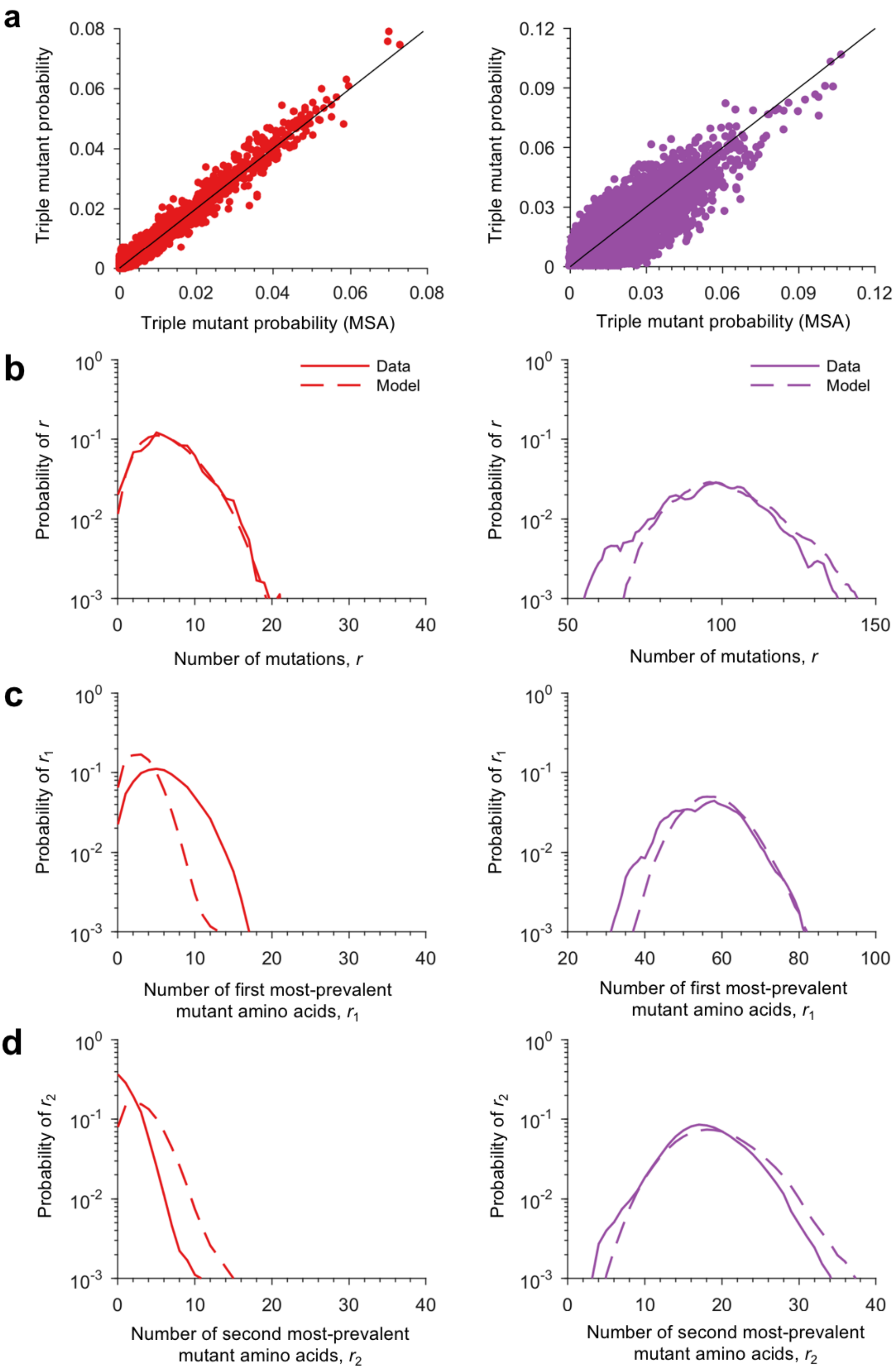

Supplementary Figure 2. Validation of the inferred Potts model for p24 (red) and gp160 (purple) proteins. Comparison of (**a**) the triple mutant probability, the distribution of the (**b**) total number of mutations, (**c**) the number of first most-prevalent mutant amino acid, and (**d**) the number of second most-prevalent mutant amino acid in the observed sequences and the inferred model.

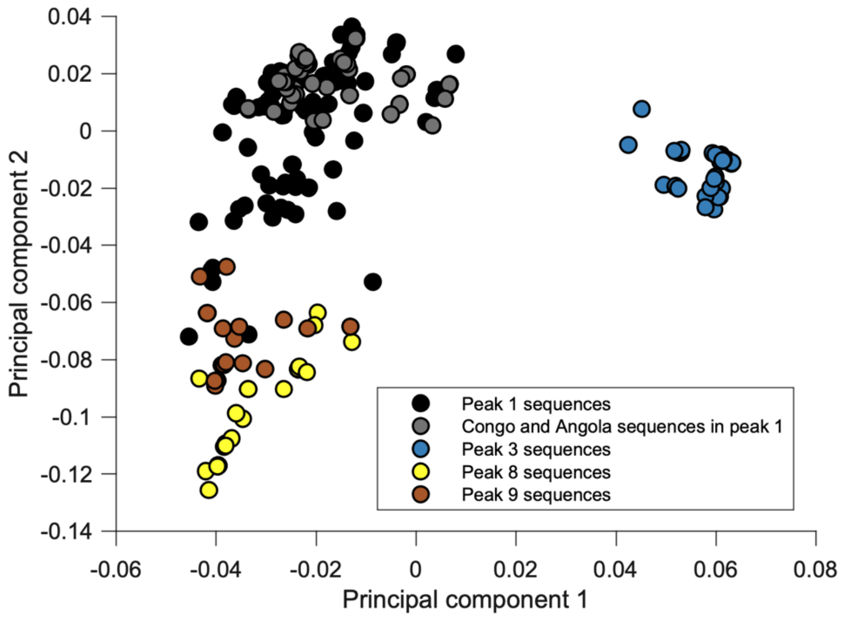

Supplementary Figure 3. Statistical analysis for comparing the similarity of Congo and Angola sequences in different peaks. The similarity between any pair of sequences was quantified using the similarity matrix (see Methods for details). Here, the similarity matrix was constructed using exclusively the sequences in peaks 1, 3, 8, and 9, as these peaks comprised Congo and Angola sequences. The scatterplot of the first two principal components of the resulting similarity matrix is shown.

a

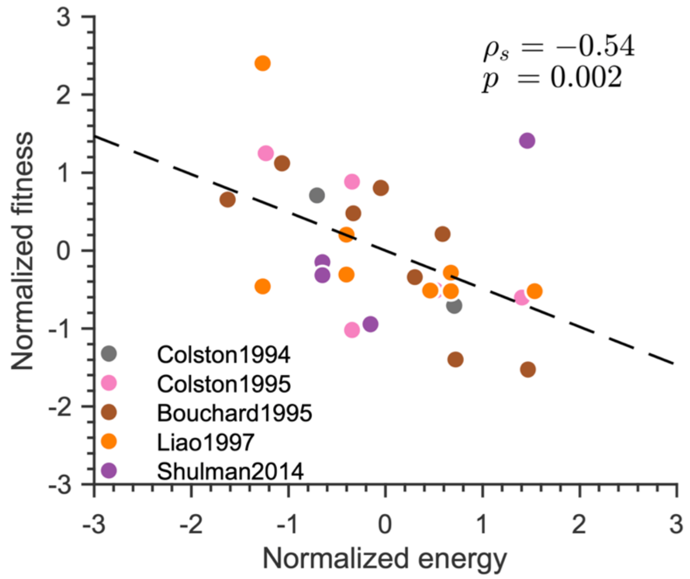

b

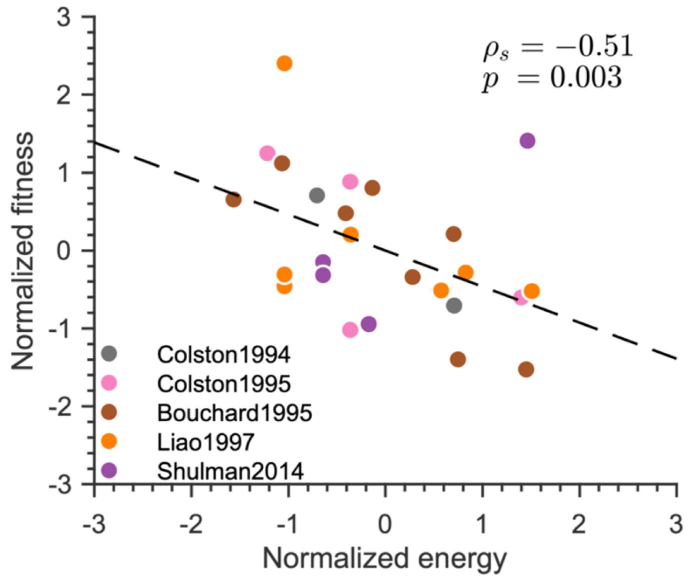

Supplementary Figure 4. Comparison of the experimental fitness measurements and in silico predicted energy values obtained from the landscapes determined using biased sequence data. Results for the landscape determined using (**a**) all sequences and (**b**) all sequences except those corresponding to peak 1. The relatively weaker Spearman correlation in (b) as compared to the landscape determined using sequences corresponding to peak 1 (Figure 5) is neither due to the lack of sequences (number of sequences in peak 1 is 484 while that in remaining peaks is 1076) nor the involved genetic diversity (mean residue entropy of peak 1 is ~0.03 while that of remaining peaks is ~0.07).

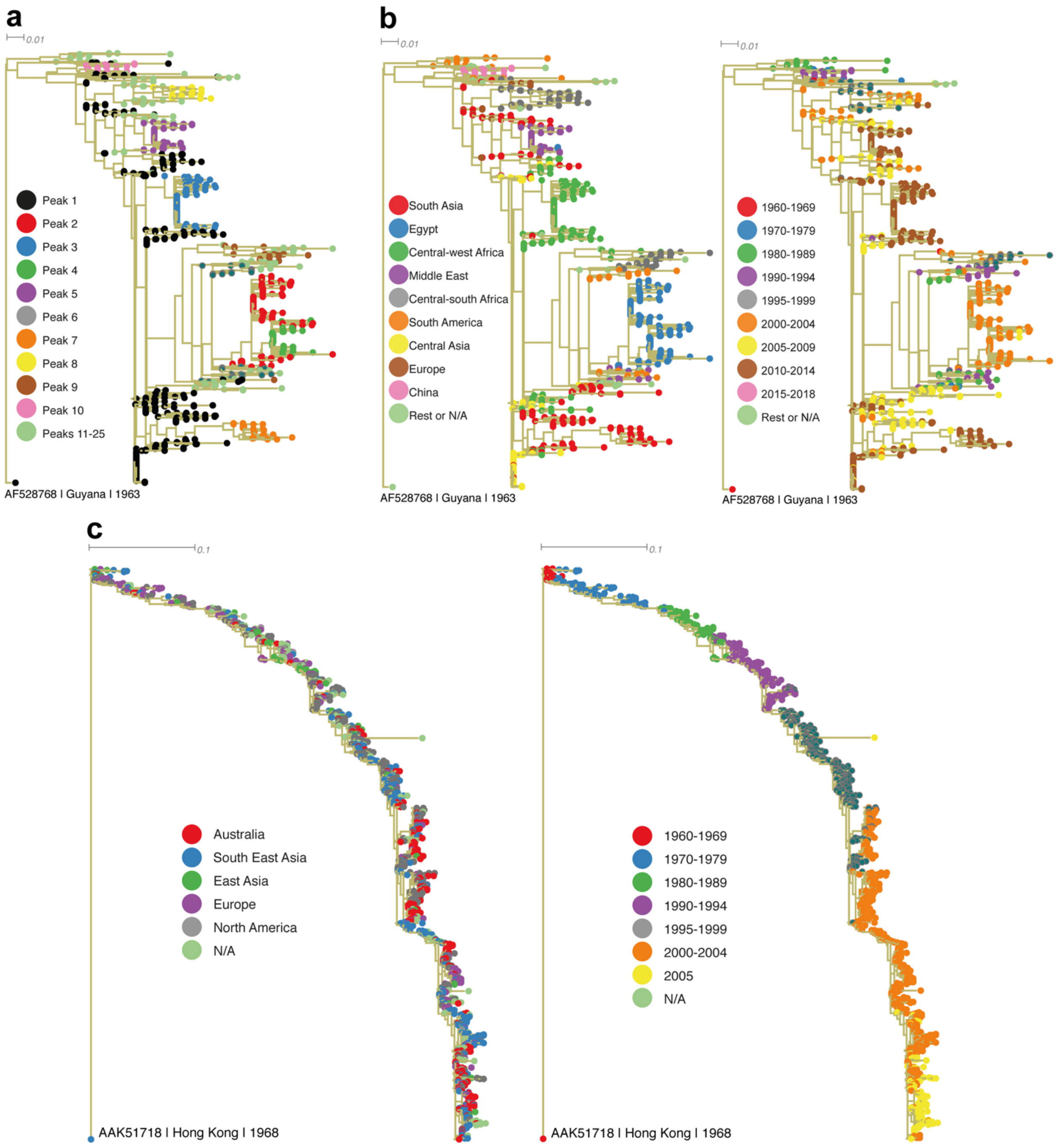

Supplementary Figure 5. Study of phylogenetic tree of vp1 and comparison with the phylogenetic tree of influenza A H3N2 hemagglutinin 1 (HA1) protein. (**a**-**b**) Rectangular phylogram of the vp1 phylogenetic tree with sequences colored according to their (**a**) peak number (similar to Fig. 6a in the main text), (**b**, *left panel*) spatial information, and (**b**, *right panel*) temporal information. (**c**) Rectangular phylogram of the HA1 phylogenetic tree with sequences colored according to (*left panel*) spatial information and (*right panel*) temporal information. It can be observed that HA1 evolves in a temporally-clustered narrowly-directed manner. This is well-known and is due to the influence of effective natural and vaccine-associated immune responses. HA1 sequences were downloaded from the NCBI database up to year 2005 to avoid sampling bias of USA-specific sequences in the recent years. All the trees were constructed using PASTA[32] and rooted with one of the oldest available sequences. All the edges were drawn with khaki color.

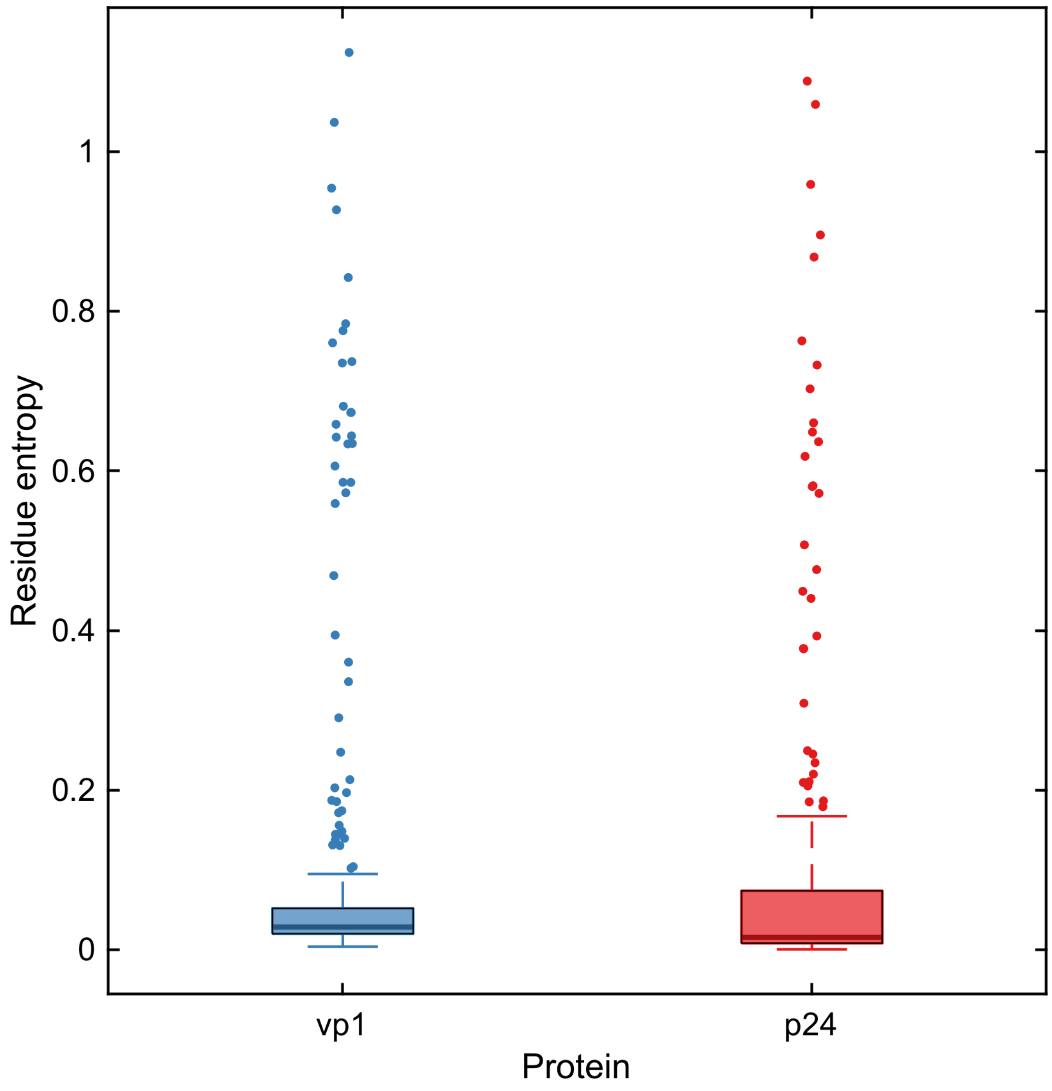

Supplementary Figure 6. Comparison of the variability of the PV and HIV capsid proteins using residue entropy. In each box plot, the horizontal line indicates the median, the edges of the box represent the first and third quartiles, and whiskers extend to span a 1.5 inter-quartile range from the edges.

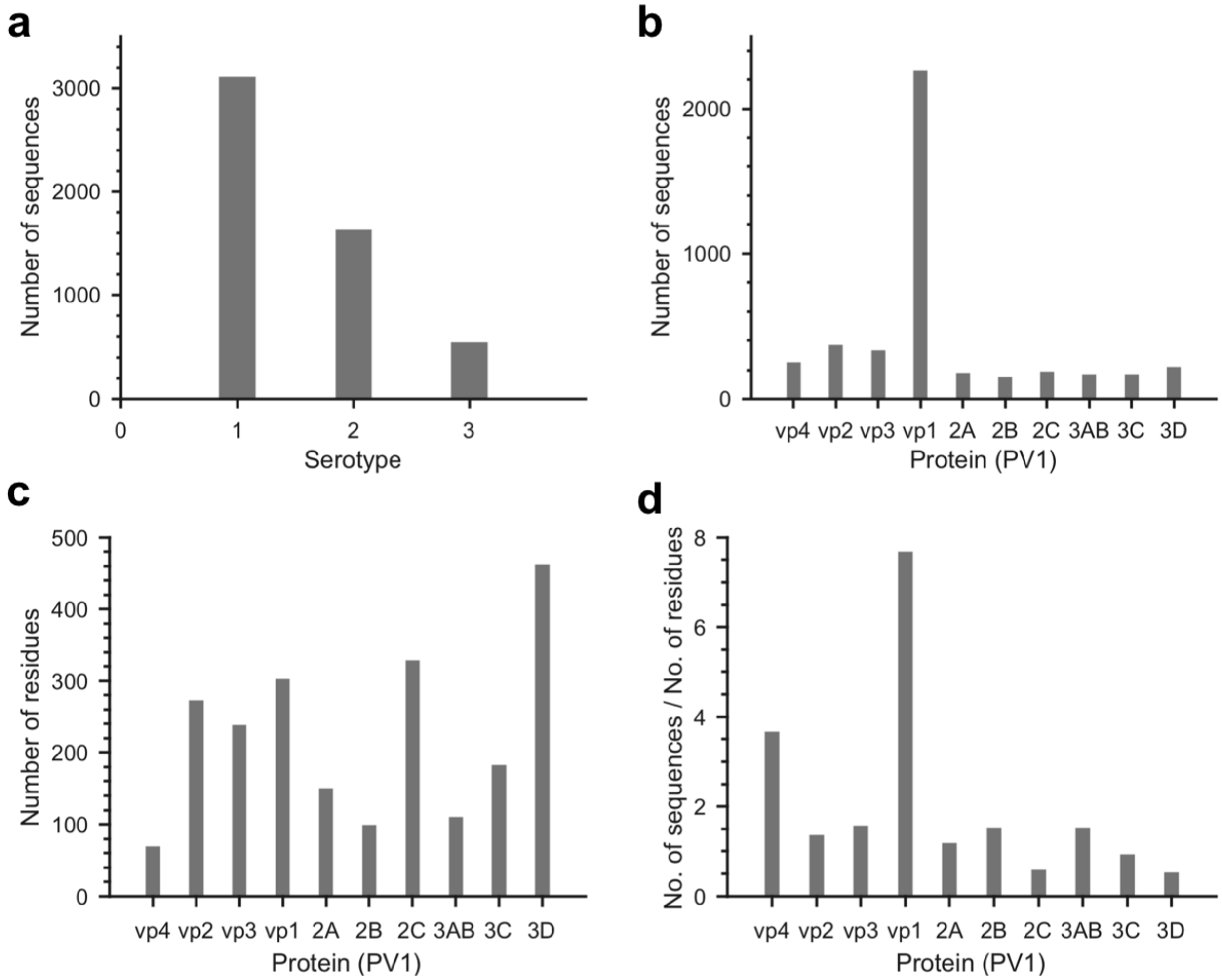

Supplementary Figure 7. Statistics of the PV sequences downloaded from the NCBI database. (**a**) Number of sequences for the three serotypes of PV, (**b**) Number of sequences for the individual proteins in PV serotype 1, (**c**) Length of each protein (Number of residues) in PV serotype 1, and (**d**) Ratio of the number of sequences to the number of residues for each protein in PV serotype 1.

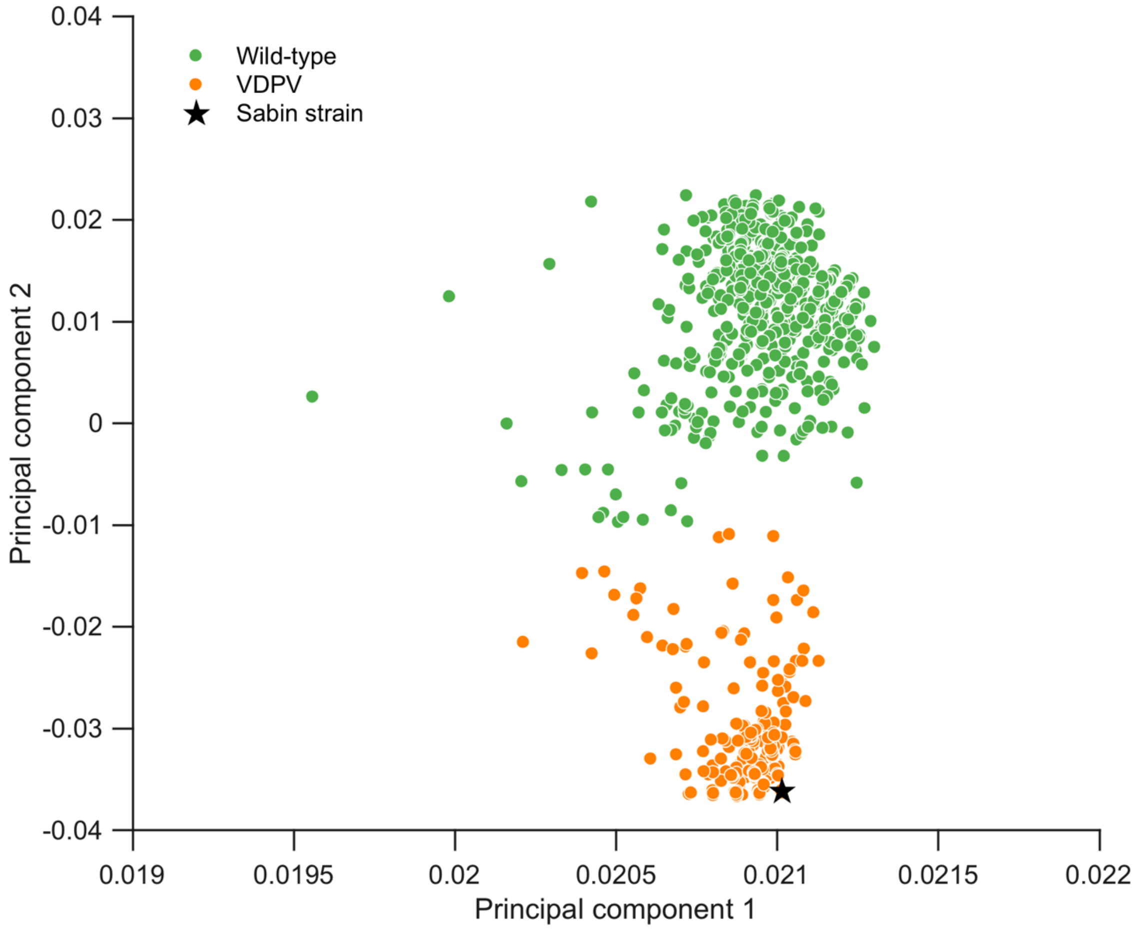

Supplementary Figure 8. Scatterplot of the first two principal components of the similarity matrix (see Methods) constructed using the available vp1 sequence data. The sequences in green and orange represent the wild-type and the VDPV sequences, respectively. The OPV strain, Sabin 1, is shown as a black star.

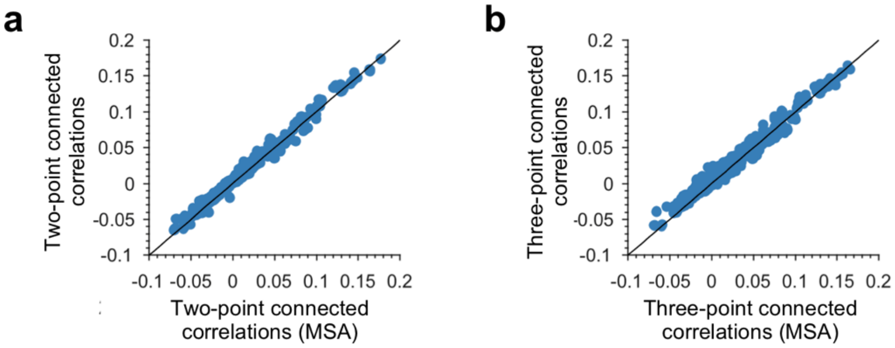

Supplementary Figure 9. The inferred Potts model of vp1 protein accurately captures the connected correlations in the data. (**a**) The two-point connected correlations, $p_{ij}(x,y) - p_i(x)p_j(y)$ and (**b**) the three-point connected correlations (which represents how mutations at two residues influence the probability of mutation at a third residue, i.e., $p_{ijk}(x,y,z) - p_i(x)p_{jk}(y,z)$), of the observed sequences and those obtained from the inferred Potts model.

a

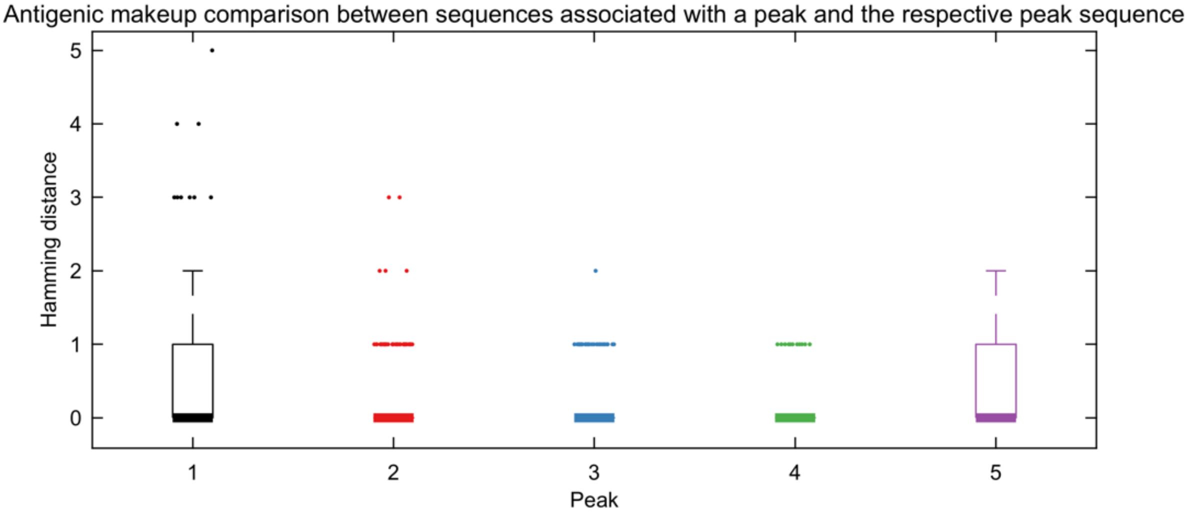

b

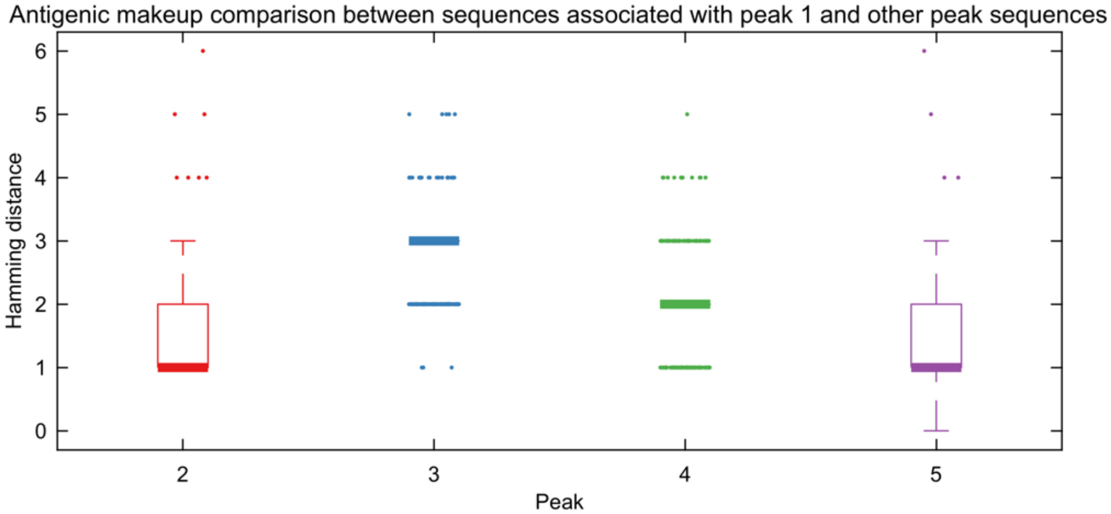

Supplementary Figure 10. The peak sequence is a good representative of the antigenic makeup of the sequences associated with that peak. In each box plot, the horizontal line indicates the median, the edges of the box represent the first and third quartiles, and whiskers extend to span a 1.5 inter-quartile range from the edges. The box plots are colored according to the scheme in Fig. 3. (**a**) The combination of amino acids at the antigenic sites in a peak sequence is shared by the majority of the sequences associated with that peak. Results are shown here for the amino acid differences, restricted to the antigenic sites, between the sequences associated with a peak and the respective peak sequence (results are shown for the top five peaks only). (**b**) The combination of amino acids at the antigenic sites in the sequences associated with a particular peak is different from that of the peak sequences representing other peaks. Results are shown here for the amino acid differences in the antigenic sites of the sequences associated with peak 1 from the peak sequences representing peaks 2 to 5.

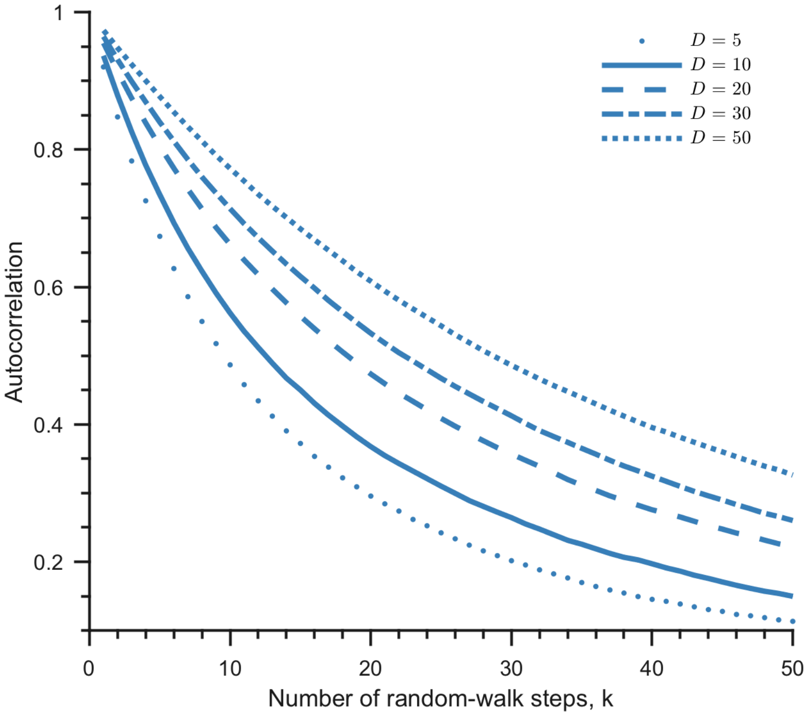

Supplementary Figure 11. The decay in the autocorrelation of energy of vp1 gets slower (implying a flatter landscape) as the Hamming distance of the starting sequences from the observed sequences (*D*) is increased.

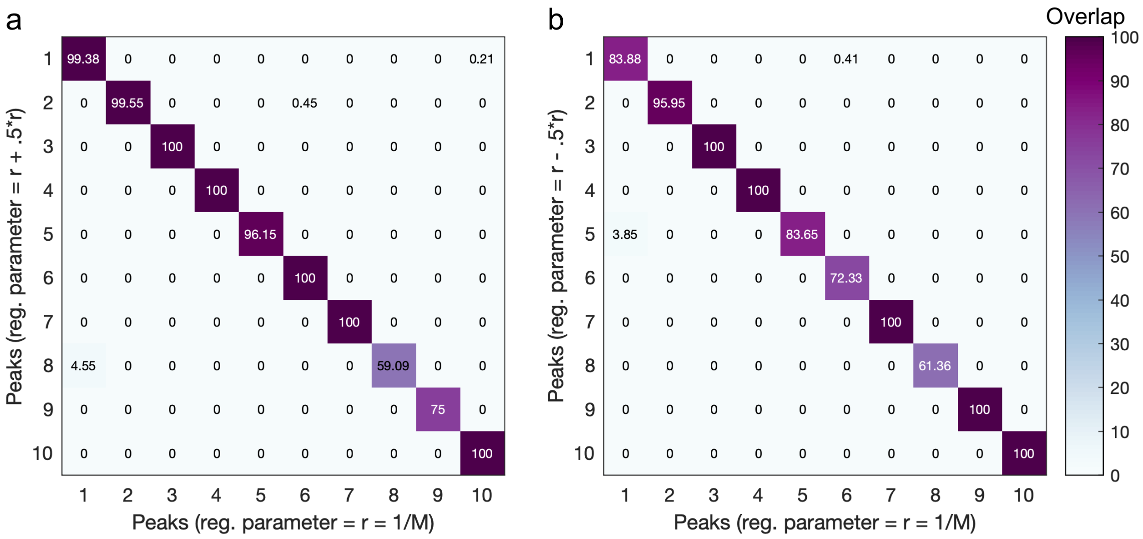

Supplementary Figure 12. Comparison of peaks obtained using different values of regularization parameter in landscape inference. Results are shown for the landscape inferred using (**a**) a 50% larger regularization parameter and (**b**) a 50% smaller regularization parameter than that one used in our landscape, respectively. The $(i,j)^{th}$ element of the matrix shows the percentage of sequences corresponding to the original peak present in the jth peak obtained using a different regularization parameter.

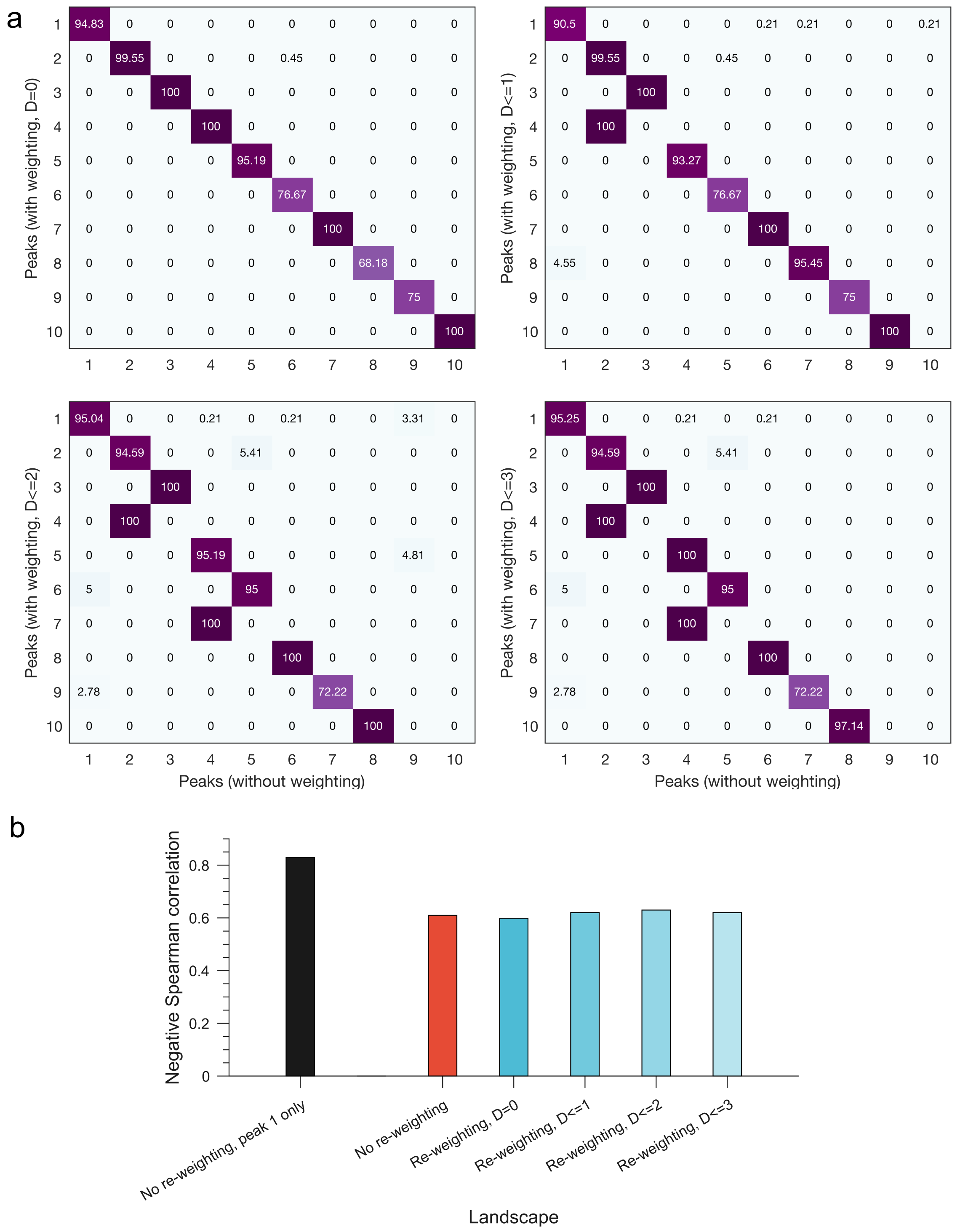

Supplementary Figure 13. Results for landscapes inferred after compensating for sampling bias via sequence reweighting. (**a**) Comparison of peaks identified in the landscapes with or without sequence re-weighting. Results are shown for the landscapes inferred using the re-weighting scheme for different values of D. The $(i,j)^{th}$ element of the matrix shows the percentage of sequences corresponding to the original peak i (identified in the landscape inferred using no re-weighting) present in the jth peak identified in the landscape inferred using re-weighting. (**b**) Comparison of the Spearman correlation obtained between the experimental fitness measurements and the predictions based on the landscapes inferred with or without the re-weighting scheme. The high correlation obtained using the landscape based on only the peak 1 sequences, proposed by us to be a meaningful representation of PV fitness landscape, is also shown for reference.

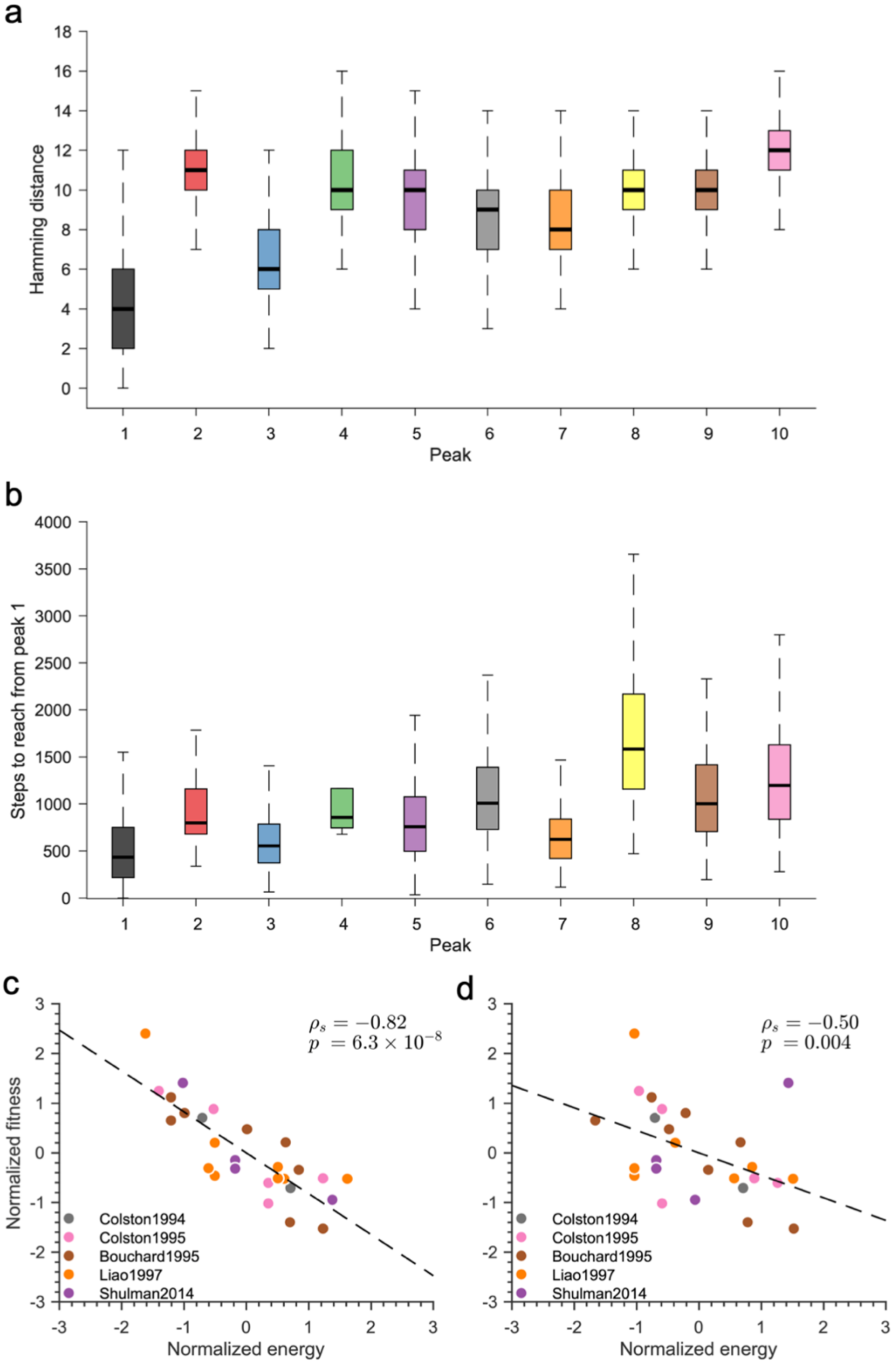

Supplementary Figure 14. Analysis of local peak 7. (**a**) Hamming distance of all sequences in each peak from sequences in peak 1. (**b**) Number of mutation steps required to reach a peak starting from sequences in peak 1. 600 zero-temperature MCMC runs were started from each sequence belonging to peak 1 and the peak reached at the end of each trajectory (total steps in each MCMC run = 5×10^5) was recorded. (**c-d**) In silico predicted energy vs the experimental fitness measurements. Both the fitness measurements and the predicted energies have been normalized using the standard procedure of subtracting the mean from each data set and dividing by its standard deviation. For [14], the virus titers reported at higher temperature of 40 degrees Celsius were used. (**c**) Comparison of the energy of the prevalence landscape inferred from the sequences corresponding to peak 1 & 7 and the experimental fitness measurements. (**d**) Comparison of the energy of the prevalence landscape inferred from the all sequences except those corresponding to peak 1 & 7 and the experimental fitness measurements. In each box plot in (a) and (b), the horizontal line indicates the median, the edges of the box represent the first and third quartiles, and whiskers extend to span a 1.5 inter-quartile range from the edges.

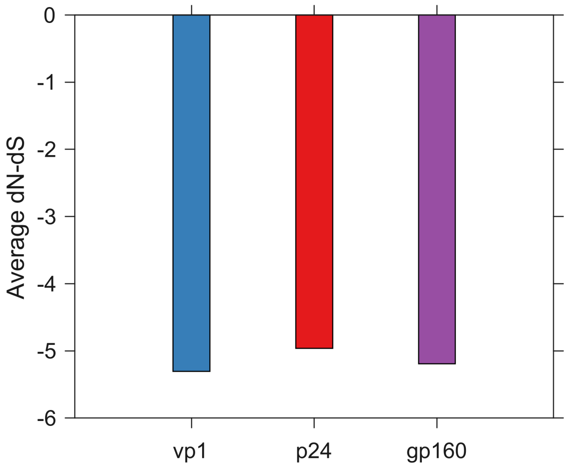

Supplementary Figure 15. Comparison of the dN-dS values obtained for each studied protein from the codon-based test of neutrality analysis between sequences. dS and dN are the numbers of synonymous and nonsynonymous substitutions per site, respectively. The average of the dN-dS values computed over all sequence pairs in a protein using the Li-Wu-Luo method[17] are shown. Similar qualitative results were obtained using another dN/dS analysis method—the modified Nei-Gojobori method. The probability of rejecting the null hypothesis of strict-neutrality (dN = dS) was set to 0.05 and only the significant dN-dS values were used to compute the average. All positions in a protein with less than 95% site coverage were eliminated. That is, fewer than 5% alignment gaps, missing data, and ambiguous bases were allowed at any position. Evolutionary analyses were conducted using the MEGA7 software[33].

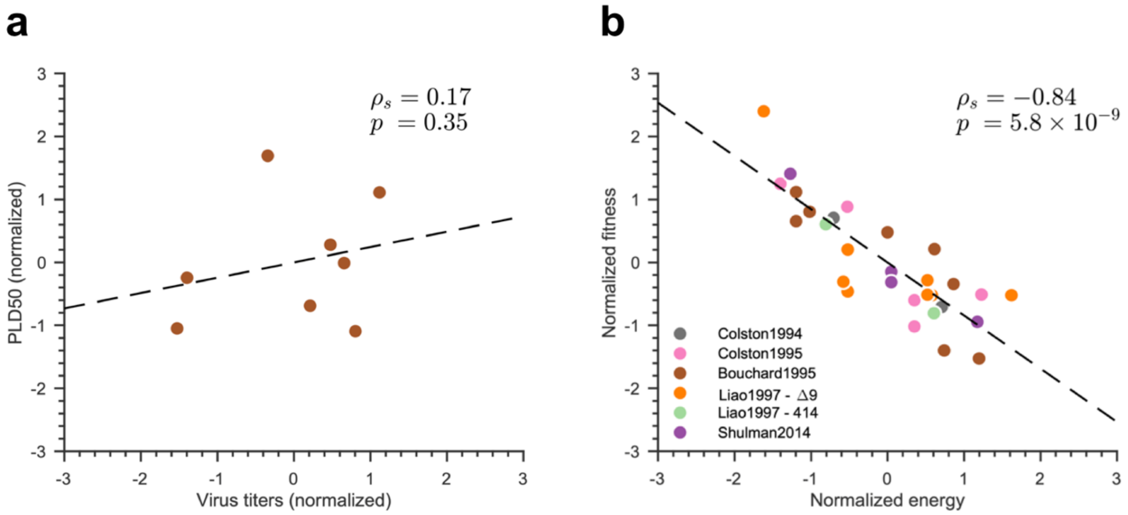

Supplementary Figure 16. Results related to the selection of experimental fitness measurements from specific reports. (**a**) Normalized virus titers vs normalized PLD50 values reported in ref[14] for the same viral strains. (**b**) Comparison of in silico predicted energy and experimental fitness measurements after including the virus titers related to the 414 strain reported in ref[13]. Both the fitness measurements and the predicted energies have been normalized using the standard procedure of subtracting the mean from each data set and dividing by its standard deviation.

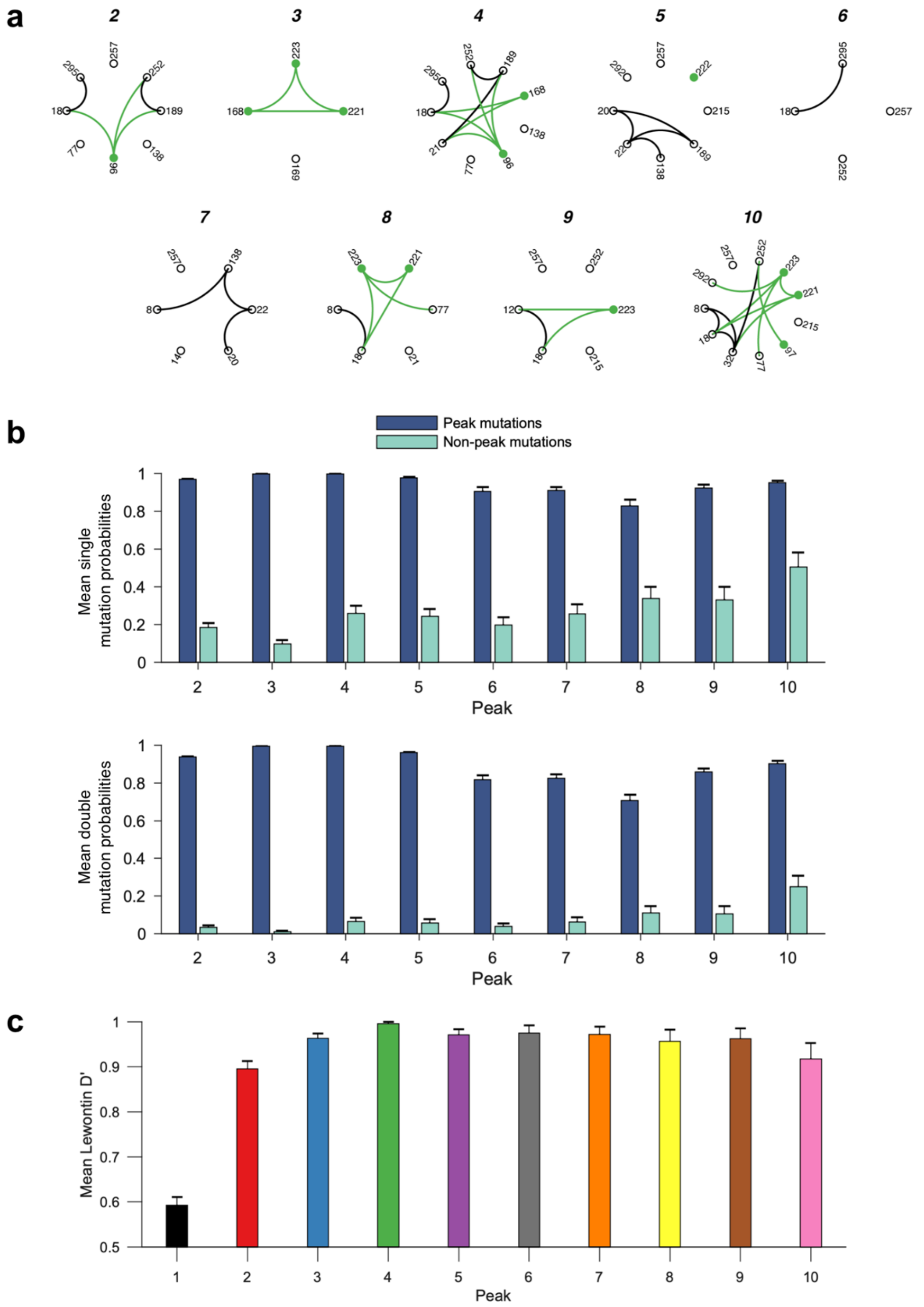

Supplementary Figure 17. Analysis of the model couplings inferred using all available sequences. (**a**) Couplings among mutations in the peak sequences are ranked among the top 1 percentile. For each peak, only the couplings among the mutations in the peak sequence were considered. The peak numbers are shown above each subfigure. The couplings that are ranked in the top 1 percentile of the inferred model couplings are shown as connected. Connections involving at least one known vp1 antigenic site are shown in green color while all remaining ones are shown in black color. Results are shown for peaks 2 to 10. (**b**) Correlations among peak mutations in each peak are stronger than the corresponding non-peak mutations. The single (top panel) and double mutation probabilities (bottom panel) were computed for each peak by considering all sequences falling in that peak. Results are shown for peaks 2 to 10. Error bars represent one standard error. (**c**) Comparison of genetic linkage associated with the sequences corresponding to each peak. Genetic linkage was quantified using the standard Lewontin D' measure. The height of each bar represents the mean D' value across all the pairs of mutations in the sequences corresponding to that peak. Error bars represent one standard error.

a

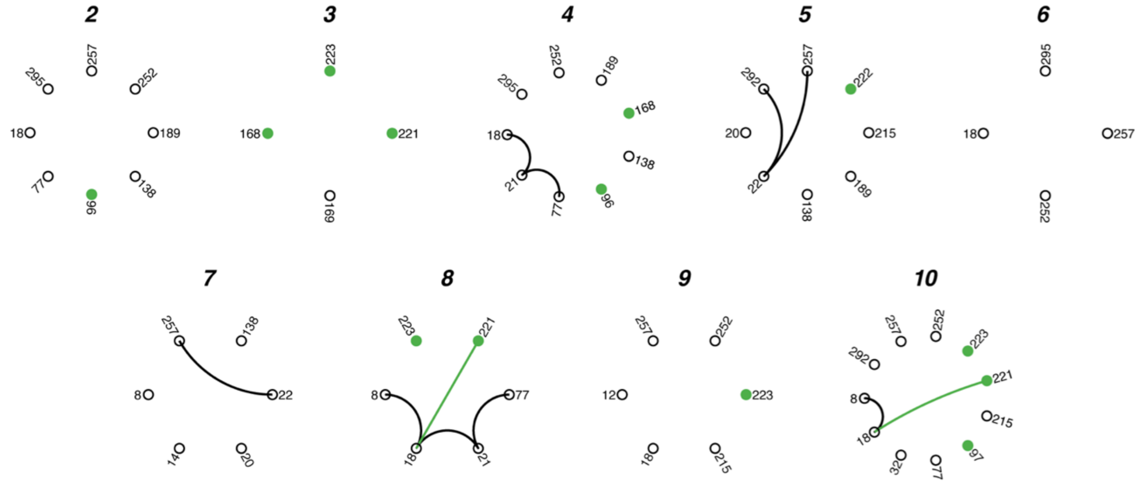

b

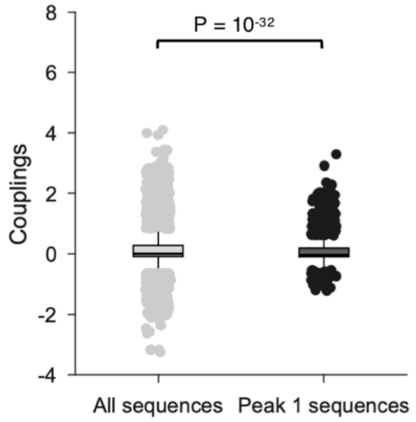

Supplementary Figure 18. Analysis of the model couplings inferred using peak 1 sequences only. (**a**) Majority of couplings among peak mutations are not ranked among the top 1 percentile of the model couplings. Note that the peaks are defined based on the all-sequence model. The peak numbers are shown above each subfigure. For each peak, only the couplings among the respective peak mutations are studied. The couplings that are ranked in the top 1 percentile of the inferred model couplings are shown as connected. The couplings between pairs involving any antigenic site are shown in green color while all remaining ones are shown in black color. Results are shown for peaks 2 to 10. (**b**) Comparison of the model couplings inferred in the all-sequence and the peak 1 based model. The magnitude of the couplings inferred in the peak 1 based model are smaller than those in the all-sequence model. In each box plot, the horizontal line indicates the median, the edges of the box represent the first and third quartiles, and whiskers extend to span a 1.5 inter-quartile range from the edges.

Supplementary Tables

Supplementary Table 1. The statistical significance of the association of each local peak with unimmunized or immunized PV-infected population.

Peak	Number of sequences				Statistical significance of association (p-value)*	
	Unimmunized population	Immunized population	Unclear/ information not available	References#	Unimmunized population	Immunized population
1	234	83	167	3,6–11	10^{-65}	NS
2	0	206	16	12-14	NS	10^{-30}
3	0	191	0	15	NS	10^{-44}
4	0	108	1	12-14	NS	10^{-23}
5	5	96	3	3,16	NS	10^{-13}
6	0	53	7	17,18	NS	10^{-6}
7	60	0	0	3	10^{-42}	NS
8	0	36	8	19	NS	10^{-3}
9	0	26	10	19	NS	10^{-1}
10	0	34	1	20	NS	10^{-7}
Total	*299*	*833*	*213*			

*The statistical significance of the association of a peak with immunized or unimmunized population was quantified as follows. Assume that there are j sequences associated with immunized (or unimmunized) population in the available data and a peak, associated with n sequences, includes i that are a subset of these j sequences of immunized (or unimmunized) population. Here, the null hypothesis would be that the observed number of sequences associated with this peak occurred from a random selection from the M available sequences. Assuming that the null hypothesis is true, the p-value is then the probability that a peak would be associated with at least i of the j sequences associated with immunized (or unimmunized) population and is also calculated using equation 8 (see Methods). A low p-value ($p < 0.05$) would indicate that the null hypothesis is rejected and that it is unlikely that a peak associated with such immunized (or unimmunized) population could arise from random chance. NS denotes the clearly non-significant ($p > 0.1$) results.

#References are numbered according to the main text.

Supplementary Table 2. Experimental fitness values reported in refs.[11–15].

(Colston & Racaniello 1994)	Virus[a]	Titer (PFU/ml)[b]
	Mahoney	4.7×10^7
	G225D	$\sim 4.4\times10^6$
	D226G	$\sim 4.4\times10^6$
	D226N	$\sim 4.4\times10^6$
	L228F	$\sim 4.4\times10^6$
	A231V	$\sim 4.4\times10^6$
	L234P	$\sim 4.4\times10^6$
	D236G	$\sim 4.4\times10^6$
	M132I	$\sim 4.4\times10^6$
	A241V	$\sim 4.4\times10^6$
	A241T	$\sim 4.4\times10^6$
	H265R	$\sim 4.4\times10^6$

(Colston & Racaniello 1995)	Virus	Titer (PFU/ml)
	Mahoney	1.6×10^9
	P95S	1.8×10^9
	V160I	7.8×10^8
	P95S, V160I	8.3×10^8

(Bouchard et al., 1995)	Virus	Titer (Plaque size)[c]
	Mahoney	0.315
	T36A	0.333
	T88A	0.125
	M90I	0.288
	P95S	0.380
	T99K	0.232
	A106T	0.348
	L134F	0.112

(Liao & Racaniello 1997)	Virus	Titer (PFU/ml)[d]
	Mahoney (Δ9)	8.5×10^8
	V160I	2.3×10^8
	W170R	4.3×10^7
	T177S	8.6×10^7
	V160I, W170R	2.6×10^7
	V160I, T177S	2.8×10^7
	W170R, T177S	9.3×10^7
	V160I, W170R, T177S	2.6×10^7

(Shulman et al., 2014)	Virus	50% paralytic dose (PD_{50})[e]
	Mahoney	5.9
	VP1_8062-PL1_ISR13	7.2
	VP1_8149-PL1_ISR13	6.7
	VP1_8150-PL1_ISR13	6.8

[a] For each site-directed mutant in the Mahoney strain, the first and last letter represents the wild-type and mutant amino acid respectively, while the number represents the position of the residue in the vp1 protein. Mutations which are not seen in the MSA were replaced with the least observed mutant at that residue to predict the energy. Note that our model is not trained to predict the energy of a strain with mutation at a fully conserved residue in the MSA. As these mutants are not present in the MSA, we can assume their fitness to be very small; the fitness of strains with these mutants was found to be smaller than the Mahoney strain in the experiments as well. Thus, we set the energy of such strains to nh_{min}, where n is the number of fully conserved residues and h_{min} is the minimum value of the fields predicted in the inferred landscape (equation 1).

[b] Only the average titers were reported for the strains with mutations in ref[15]. The corresponding predicted energies were also averaged in the comparison in Fig. 5a.

[c] Instead of PFU/ml, the viral growth in ref[14] was reported in terms of plaque size. The values reported for 40 degrees Celsius were used. Note that this choice of measure of viral growth is irrelevant for our analysis as we are interested in the relative growth of mutant strains with respect to the reference strain used in each report.

[d] Instead of Mahoney strain, Δ9—a B-C loop (residues 98–102) truncated Mahoney strain—was used in ref[13].

[e] Only those strains were included in the analysis for which accession number was provided in ref[11]. The PD_{50} value reported for the Sabin 1 strain was excluded as our model is specific to the wild-type PV. In contrast to the viral titers and growth data from other reports, the smaller the PD50 value, the more is the virus's virulence. Thus, for consistency, we used the inverse of the reported values of PD50.

Supplementary References

1. Barton, J. P., Kardar, M. & Chakraborty, A. K. Scaling laws describe memories of host–pathogen riposte in the HIV population. *Proc. Natl. Acad. Sci.* **112**, 1965–1970 (2015).

2. Barton, J. P., De Leonardis, E., Coucke, A. & Cocco, S. ACE: adaptive cluster expansion for maximum entropy graphical model inference - biorxiv. *Bioinformatics* **32**, 3089–3097 (2016).

3. Morcos, F. *et al.* Direct-coupling analysis of residue coevolution captures native contacts across many protein families. *Proc. Natl. Acad. Sci. U. S. A.* **108**, E1293–E1301 (2011).

4. Cocco, S., Monasson, R. & Weigt, M. From principal component to direct coupling analysis of coevolution in proteins: Low-eigenvalue modes are needed for structure prediction. *PLoS Comput. Biol.* **9**, e1003176 (2013).

5. El Bassioni, L. *et al.* Prolonged detection of indigenous wild polioviruses in sewage from communities in Egypt. *Am. J. Epidemiol.* **158**, 807–815 (2003).

6. Drexler, J. F. *et al.* Robustness against serum neutralization of a poliovirus type 1 from a lethal epidemic of poliomyelitis in the Republic of Congo in 2010. *Proc. Natl. Acad. Sci. U. S. A.* **111**, 12889–94 (2014).

7. Shulman, L. M. *et al.* Resolution of the pathways of poliovirus type 1 transmission during an outbreak. *J. Clin. Microbiol.* **38**, 945–952 (2000).

8. Jorba, J., Campagnoli, R., De, L. & Kew, O. Calibration of multiple poliovirus molecular clocks covering an extended evolutionary range. *J. Virol.* **82**, 4429–4440 (2008).

9. Odoom, J. K. *et al.* Interruption of poliovirus transmission in Ghana: Molecular epidemiology of wild-type 1 poliovirus isolated from 1995 to 2008. *J. Infect. Dis.* **206**, 1111–1120 (2012).

10. Shaukat, S. *et al.* Molecular characterization and phylogenetic relationship of wild type 1 poliovirus strains circulating across Pakistan and Afghanistan bordering areas during 2010–2012. *PLoS One* **9**, e107697 (2014).

11. Shulman, L. M. *et al.* Genetic analysis and characterization of wild poliovirus type 1 during sustained transmission in a population with 95% vaccine coverage, Israel 2013. *Clin. Infect. Dis.* **60**, 1057–1064 (2015).

12. Colston, E. M. & Racaniello, V. R. Poliovirus variants selected on mutant receptor-expressing cells identify capsid residues that expand receptor recognition. *J. Virol.* **69**, 4823–4829 (1995).

13. Liao, S. & Racaniello, V. Allele-specific adaptation of poliovirus VP1 B-C loop variants to mutant cell receptors. *J. Virol.* **71**, 9770–9777 (1997).

14. Bouchard, M. J., Lam, D. & Racaniello, V. R. Determinants of attenuation and temperature sensitivity in the type 1 poliovirus Sabin vaccine. *J. Virol.* **69**, 4972–4978 (1995).

15. Colston, E. & Racaniello, V. R. Soluble receptor-resistant poliovirus mutants identify surface and internal capsid residues that control interaction with the cell receptor. *EMBO J.* **13**, 5855–5862 (1994).

16. Martin, J. *et al.* Long-term excretion of vaccine-derived poliovirus by a healthy child. *J. Virol.* **78**, 13839–13847 (2004).

17. Li, W.-H., Wu, C.-I. & Luo, C.-C. A new method for estimating synonymous and

nonsynonymous rates of nucleotide substitution considering the relative likelihood of nucleotide and codon changes. *Mol. Biol. Evol.* **2**, 150--174 (1985).

18. Kryazhimskiy, S. & Plotkin, J. B. The population genetics of dN/dS. *PLoS Genet.* **4**, e1000304 (2008).

19. Ferguson, A. L. *et al.* Translating HIV sequences into quantitative fitness landscapes predicts viral vulnerabilities for rational immunogen design. *Immunity* **38**, 606–617 (2013).

20. Mann, J. K. *et al.* The fitness landscape of HIV-1 Gag: Advanced modeling approaches and validation of model predictions by in vitro testing. *PLoS Comput. Biol.* **10**, e1003776 (2014).

21. Chakraborty, A. K. & Barton, J. P. Rational design of vaccine targets and strategies for HIV: a crossroad of statistical physics, biology, and medicine. *Reports Prog. Phys.* **80**, 032601 (2017).

22. Louie, R. H. Y., Kaczorowski, K. J., Barton, J. P., Chakraborty, A. K. & McKay, M. R. Fitness landscape of the human immunodeficiency virus envelope protein that is targeted by antibodies. *Proc. Natl. Acad. Sci.* **115**, E564–E573 (2018).

23. Barton, J. P. *et al.* Relative rate and location of intra-host HIV evolution to evade cellular immunity are predictable. *Nat. Commun.* **7**, 11660 (2016).

24. Butler, T. C., Barton, J. P., Kardar, M. & Chakraborty, A. K. Identification of drug resistance mutations in HIV from constraints on natural evolution. *Phys. Rev. E* **93**, 1–8 (2016).

25. Neverov, A. D., Kryazhimskiy, S., Plotkin, J. B. & Bazykin, G. A. Coordinated evolution of influenza A surface proteins. *PLOS Genet.* **11**, e1005404 (2015).

26. Strelkowa, N. & Lässig, M. Clonal interference in the evolution of influenza. *Genetics* **192**, 671–82 (2012).

27. Slatkin, M. Linkage disequilibrium--understanding the evolutionary past and mapping the medical future. *Nat. Rev. Genet.* **9**, 477–485 (2008).

28. Lewontin, R. C. The interaction of selection and linkage. I. General considerations; heterotic models. *Genetics* **49**, 49–67 (1964).

29. Burch, C. L., Turner, P. E. & Hanley, K. A. Patterns of epistasis in RNA viruses: A review of the evidence from vaccine design. *J. Evol. Biol.* **16**, 1223–1235 (2003).

30. de Visser, J. A. G. M. & Elena, S. F. The evolution of sex: empirical insights into the roles of epistasis and drift. *Nat. Rev. Genet.* **8**, 139–149 (2007).

31. Hogle, J. M. Poliovirus Cell Entry: Common Structural Themes in Viral Cell Entry Pathways. *Annu. Rev. Microbiol.* **56**, 677–702 (2002).

32. Mirarab, S. *et al.* PASTA: Ultra-large multiple sequence alignment for nucleotide and amino-acid sequences. *J. Comput. Biol.* **22**, 377–386 (2015).

33. Kumar, S., Stecher, G. & Tamura, K. MEGA7: Molecular Evolutionary Genetics Analysis Version 7.0 for Bigger Datasets. *Mol. Biol. Evol.* **33**, 1870–1874 (2016).
