# Supplementary material for: Deconvolving mutational patterns of poliovirus outbreaks reveals its intrinsic fitness landscape
Source: Nat Commun. 2020 Jan 17;11:377. doi: 10.1038/s41467-019-14174-2 (PMC6969152; doi:10.1038/s41467-019-14174-2)
Supplement: Supplementary file 3 — Description of Additional Supplementary Files [file 41467_2019_14174_MOESM3_ESM.pdf]

Description of Additional Supplementary Files

File Name: Supplementary Data 1

Description: The detailed metadata compiled for vp1 sequences from the NCBI database and literature.
